# Continuous Flow Synthesis of α-Trifluoromethylthiolated Esters and Amides from Carboxylic Acids: a Telescoped Approach

**DOI:** 10.1021/acs.joc.1c01270

**Published:** 2021-07-27

**Authors:** Francesca Franco, Sara Meninno, Alessandra Lattanzi, Alessandra Puglisi, Maurizio Benaglia

**Affiliations:** † Dipartimento di Chimica e Biologia “A. Zambelli”, Università di Salerno, Via Giovanni Paolo II, 84084 Fisciano, Italy; ‡ Dipartimento di Chimica, Università degli Studi di Milano, Via Golgi 19, 20133 Milano, Italy

## Abstract

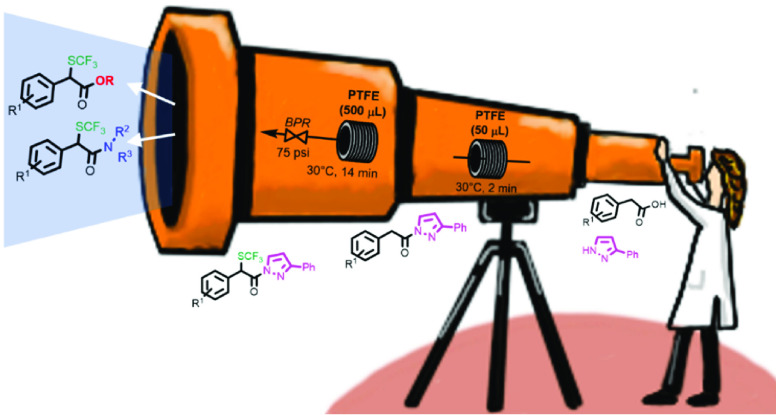

A continuous flow approach to access α-trifluoromethylthiolated esters and amides using commercially available arylacetic acids and *N*-(trifluoromethylthio)phthalimide as the electrophilic reagent is described. The experimental protocol involves the in-flow conversion of the carboxylic acid into *N-*acylpyrazole followed by the α-trifluoromethylthiolation in a PTFE coil reactor and final reaction with primary or secondary amines, or alcohols, to afford in a telescoped process α-substituted SCF_3_ amides and esters, respectively, in good overall yield and short reaction times.

The growing interest in academia and industrial context, toward effective and large-scale syntheses of intermediates and active pharmaceutical ingredients (APIs), has seen the flourishing of flow chemistry as a powerful enabling technology.^1^ An efficient optimization of the single transformations of a multistep synthesis, under better controlled and mild conditions, may lead to the development of telescoped processes, thus avoiding isolation of intermediates and maximizing efficiency, practicality, and environmental impact.^2^

Fluorinated compounds find important applications in the pharmaceutical and agrochemical industries.^3^ Indeed, when the polar C–F bond or fluorine-containing groups are present, the pharmacokinetics, lipophilicity, and bioavailability of the molecule is significantly affected and then can be modulated.^4^ Accordingly, over the past decades, many investigations have been undertaken to develop methods useful for installing fluorine-containing groups onto organic molecules.^5^

We recently reported the α-trifluoromethylthiolation of *N*-acyl pyrazoles using *N*,*N*,*N’*,*N’*-tetramethyl-1,8-naphthalenediamine (proton sponge) as a catalytic base and *N*-(trifluoromethylthio)phthalimide as the electrophilic source.^6^*N*-Acylpyrazoles are readily available carboxylic acid surrogates,^7^ which were suitable for undergoing α-functionalization via enolate formation under mild basic conditions (Scheme 1). In the same pot, the pyrazole group has been easily replaced by different nucleophiles, such as alcohols, amines, and water, to give the formal α-trifluoromethylthiolation of carboxylic acid derivatives. Although this methodology enabled us to successfully obtain the α-trifluoromethythiolated esters, amides, carboxylic acids, and other useful derivatives, the *N*-acyl pyrazoles had to be first synthesized from commercially available aryl/heteroaryl acetic acids. To make the entire process more convenient and practical, we envisaged that a telescoped continuous flow protocol,^8^ starting directly from the commercially and readily available carboxylic acids, would have improved the method further (Scheme 1).^9^

**Scheme 1:**
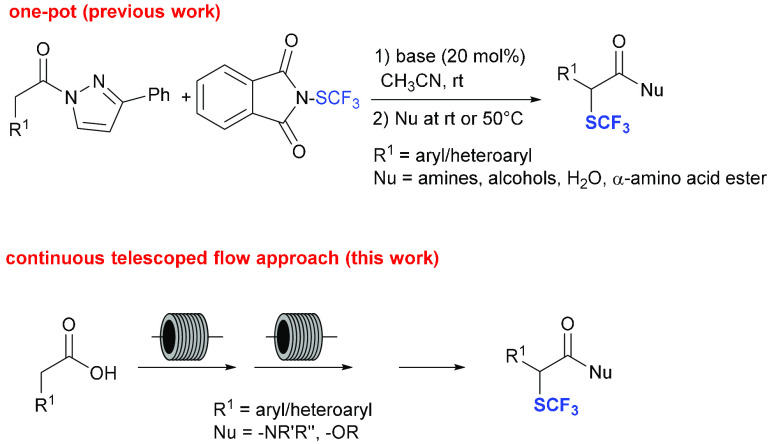
Batch and Continuous-Flow Approaches to the Synthesis of α-Trifluoromethylthio Carboxylic Acid Derivatives via *N*-Acylpyrazoles

We started our investigation by studying the conversion of the carboxylic acid into the corresponding *N-*acylpyrazole under continuous flow conditions using a PTFE coil reactor (1.58 mm outer diameter, 0.78 mm inner diameter) coiled in a bundle and immersed in an oil bath heated to the desired temperature. A 2.5 mL SGE gastight syringe A, containing phenylacetic acid and DMAP (0.1 mol/equiv) and a syringe B, containing 3 phenylpyrazole (0.63 M solution) and EDC*HCl (1.2 mol/eq), were connected by a PEEK tee junction to a 500 μL PTFE coil reactor. In some explorative tests different solvents and flow rates were studied, and a few selected results are reported in Table 1.

**Table 1:**
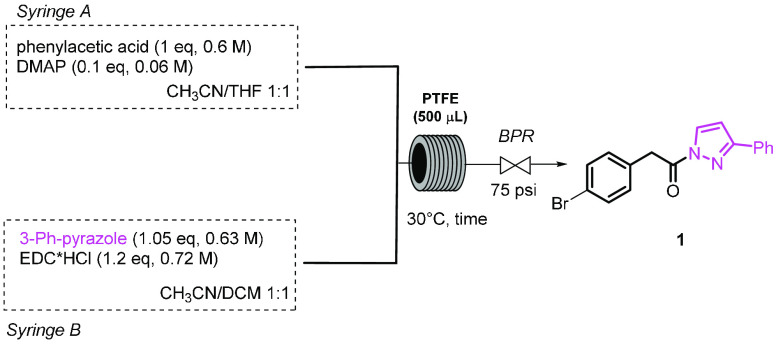
In Flow Synthesis of *N*-Acylpyrazoles^a^

entry	solvent	flow rate (mL/min)	resid, time (min)	NMR yield^b^ (%)
1	DCM	0.1	5	94
2	DCM	0.25	2	95
3	DCM	1.0	0.5	85
4	THF/CH_3_CN/DCM	0.25	2	92
5^c^	THF/CH_3_CN/DCM	0.25	2	93

a Reaction conditions: concentration of 0.1 M carboxylic acid.

b Yield determined by ^1^H NMR analysis of crude reaction with 1,3,5-trimethoxybenzene as an internal standard.

c Reaction run at a concentration of 0.3 M carboxylic acid.

After only 2 min of residence time, product **1** was obtained in DCM in 95% yield. However, in view of the following steps in the continuous process, other solvent systems were investigated, and among them the THF/CH_3_CN/DCM mixture was found to be optimal also in the preparation of the *N-*acylpyrazole (92% yield with 2 min residence time). Having established the conditions for a high yielding preparation of the activated ester, the trifluoromethylthiolation step was further investigated (Scheme 2).

**Scheme 2:**
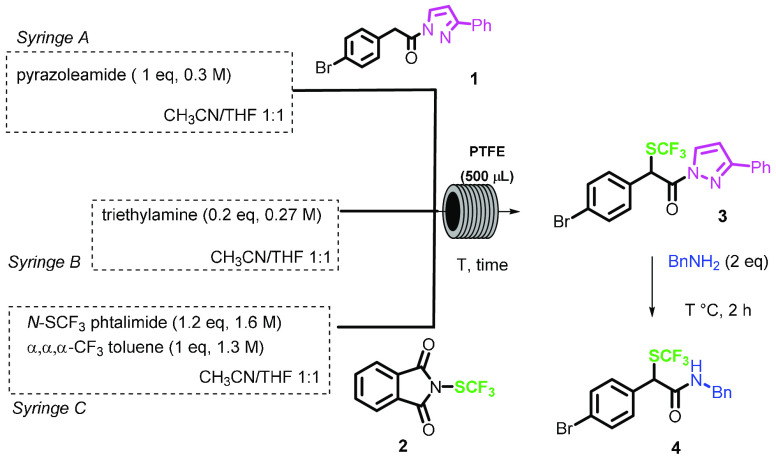
Synthesis of α-Trifluoromethylthioamides from *N*-Acylpyrazoles under Continuous Flow Conditions

A 5 mL SGE gastight syringe, containing the pyrazoleamide of 4-bromophenylacetic acid **1** as a model substrate (3 mL of a 0.3 M solution in CH_3_CN/THF 1:1, 1.0 equiv) and two 1 mL SGE gastight syringes, containing a base (0.27 M solution in CH_3_CN/THF 1:1, 0.2 equiv) and *N*-SCF_3_ phthalimide **2** (1.6 M solution in CH_3_CN/THF 1:1, 1.2 equiv), respectively, were connected by a PEEK tee junction to a 500 μL PTFE coil reactor. The outcome of the reactor, the pyrazole derivative **3**, was collected in a vial containing benzylamine (2 equiv), where it was stirred for further 2 h to afford *N*-benzyl amide **4**. The yield was evaluated by ^19^F NMR and confirmed after chromatographic purification. At first, proton sponge (PS) was evaluated as a base, since it proved to be the base of election in the reaction.^6^ However, low yields were observed, due to solubility issues (Table 2, entry 1–4); therefore, other conditions were investigated and the combination of triethylamine with acetonitrile/THF mixture was found to be a good solution and allowed to perform the reaction in continuo in 61% yield and 30 min residence time (entry 5). Higher productivity can be obtained by further increasing the flow rate (entry 6, productivity = 107.4 mg/h), and better yields were achieved by operating at 60 °C (75% yield, entry 8 of Table 2).

**Table 2:** Synthesis of *N*-Benzyl α-Trifluoromethylthioamide from *N*-Acylpyrazoles under Flow Conditions^a^

entry	solvent	base	*T* (°C)	resid time (min)	yield^b^ (%)
1	THF	PS	25	30	0
2	THF	PS	25	15	0
3	THF/CH_3_CN (1:1)	PS	30	30	20
4	THF/CH_3_CN (1:1)	PS	30	15	15
5	THF/CH_3_CN (1:1)	TEA	30	30	61
6	THF/CH_3_CN (1:1)	TEA	30	15	41
7	THF/CH_3_CN (1:1)	TEA	45	15	52 (51)
8	THF/CH_3_CN (1:1)	TEA	60	15	75 (73)
9	THF/CH_3_CN (1:1)	TEA	90	15	50
10^c^	THF/CH_3_CN (1:1)	TEA	45	15	83
11^d^	THF/CH_3_CN (1:1)	TEA	45	15	85 (83)

a Reaction conditions: 0.2 mol/equiv of TEA was used.

b Yield determined by ^19^F NMR analysis of the crude reaction product with α,α,α-CF_3_C_6_H_5_ as internal standard and in parentheses as yield after chromatographic purification.

c 0.35 mol/equiv of TEA.

d 0.5 mol/equiv of TEA.

By further optimization work, where reaction temperature, base stoichiometry, and flow rates were studied, 45 °C was identified as the ideal reaction temperature, and 0.35 or 0.5 mol/equiv of TEA, and 15 min of residence time for a 0.5 mL PTFE coil reactor as good experimental set up (85% yield, entries 10 and 11, Table 2 (see the Experimental Section for further details) affording a productivity = 217.4 mg/h of product **3**. For the sake of comparison, the in-batch reaction,^6^ performed under the same experimental conditions (THF/CH_3_CN (1:1), TEA), afforded 66 mg of product **3** after 7h reaction time, while the same amount was produced in flow after 20 min. In order to proof the scalability of the method, the reaction was conducted using the same 0.5 mL PTFE reactor for 10 h under the optimized conditions of entry 10, Table 2, affording 2.02 g of *N-*benzyl amide **4**.

The key step of the trifluoromethylthiolation was briefly investigated also with microreactor technology by using a 10 μL glass microreactor, observing a similar behavior and dependence of the yield from the temperature profile (Table 3). Operating under the same experimental conditions, in CH_3_CN–THF mixture and with TEA as a base, 65% conversion for product **4** was obtained at 60 °C with 3.3 min of residence time and 50% conversion with 1.3 min residence time (*P* = 14.3 mg/h for entry 2, *P* = 28 mg/h for entry 6).

**Table 3:**
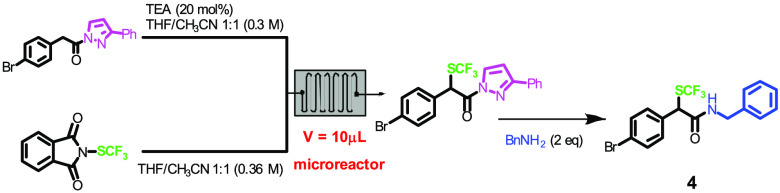
Synthesis of α-Trifluoromethylthio Amide **4** from *N*-Acylpyrazoles in Microreactors^a^

entry	*T* (°C)	flow rate (μL/min)	resid time (min)	NMR yield^b^ (%)
1	45	3	3.3	57
2	60	3	3.3	65
3	90	3	3.3	56
4	30	7.5	1.3	43
5	45	7.5	1.3	45
6	60	7.5	1.3	50
7	90	7.5	1.3	46

a 0.2 molar equiv of TEA was used.

b Yield determined by ^19^F NMR analysis of the crude reaction with α,α,α-CF_3_C_6_H_5_ as internal standard.

Finally, the telescoped process was studied (Scheme 3).

**Scheme 3:**
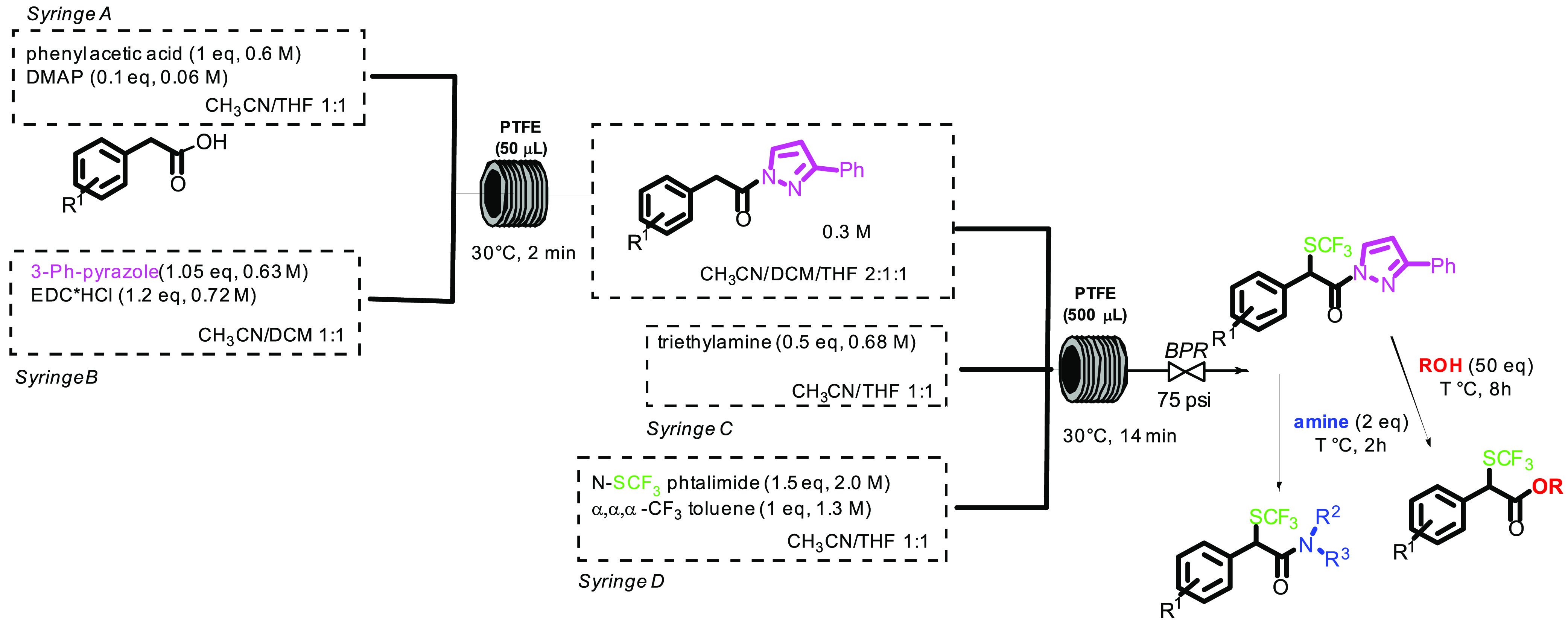
Telescopic Synthesis of α-Trifluoromethylthio Amides and Esters from Carboxylic Acids

The condensation of the arylacetic acid with pyrazole was performed in a 50 μL PTFE coil reactor (2 min residence time), and then the outcome of the reactor was connected to another tee junction, fed by two 2.5 mL SGE gastight syringes, containing TEA and *N*-SCF_3_ phthalimide, that were reacted at 30 °C in a second coil reactor (0.5 mL, 14 min residence time). After the first volume was discarded, the outcome of the reactor was collected in a vial containing amine (2 equiv), where it was stirred for further 2 h (Table 4).

**Table 4:** Telescoped Synthesis of α-Trifluoromethylthio *N*-Benzylamide **4** from 4-Bromophenylacetic Acid

entry	**2** (molar equiv)	base (molar equiv)	*T* (°C)	yield^a^ (%)
1	1.2	0.2	30	30
2	1.2	0.5	30	58 (56)
3	1.5	0.5	30	72 (70)
4	1.5	0.45	60	65
5	1.5	0.5	60	55
6	1.5	0.35	60	52
7^b^	1.5	0.5	60	50 (48)

a Yield determined by ^19^F NMR analysis of crude reaction with α,α,α-CF_3_C_6_H_5_ as internal standard and in parentheses as the yield after chromatographic purification.

b Residence time second reactor: 7 min.

Some further optimization studies were necessary in order to set up an efficient multistep, telescopic process. Using a little excess of *N*-SCF_3_ phthalimide (1.5 mol/equiv) and operating at 30 °C, 70% overall yield starting from the carboxylic acid was reached (*P* = 183.4 mg/h of product **4** entry 3).

Under the same experimental conditions, the multistep synthesis in flow of other amides was successfully achieved, in overall good yields (Figure 1).

**Figure 1:**
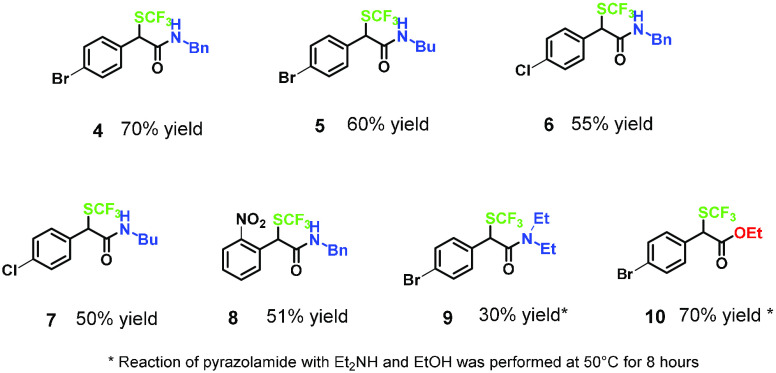
Telescoped synthesis of α-trifluoromethylthioamides and esters from carboxylic acids.

Starting from the carboxylic acid, the protocol allowed to synthesize α-trifluoromethylthio *N-*benzyl and *N-*alkyl amides *in continuo* in overall yields, ranging from 50 to 70%. The *N*,*N*-dialkyl amide **9** was obtained in lower yield, likely ascribed to more sterically hindered nature of the amine, whereas the preparation of an ester derivative **10** was satisfactorily accomplished in 70% yield. In both cases, longer reaction times for the acyl substitution were necessary.

In conclusion, a telescopic process for the synthesis of α-trifluoromethylthiolated esters and amides, using commercially available arylacetic acids and *N*-(trifluoromethylthio)phthalimide, was successfully accomplished. Micro- and mesoreactor technology was successfully employed to develop a multistep protocol for the in-flow synthesis of fluorinated derivatives in good yields and short reaction times, compared to the in-batch reactions. Based on the high versatility of the *N-*acylpyrazoles as activated carboxylic acids, the methodology is expected to offer further opportunities for the development of many other telescopic processes to access a wide variety of useful derivatives.

## Experimental Section

### General Methods

All reactions requiring dry or inert conditions were conducted in flame-dried glassware under a positive pressure of nitrogen. Anhydrous THF, dicloromethane, and acetonitrile were purchased from Aldrich. Reactions were monitored by thin-layer chromatography (TLC) on Macherey-Nagel precoated silica gel plates (0.25 mm) and visualized by UV light. Flash chromatography was performed on Merck silica gel (60, particle size: 0.040–0.063 mm). ^1^H, ^13^C, and ^19^F NMR spectra were recorded on a Bruker Avance III HD 600, Bruker Avance-400, Bruker Avance-300, or Bruker Avance-250 spectrometer in CDCl_3_ as solvent at room temperature. Chemical shifts for protons are reported using residual solvent protons (^1^H NMR: δ = 7.26 ppm for CDCl_3_) as internal standard. Carbon spectra were referenced to the shift of the ^13^C signal of CDCl_3_ (δ = 77.0 ppm). The following abbreviations are used to indicate the multiplicity in NMR spectra: s, singlet; d, doublet; t, triplet; q, quartet; dd, double doublet; ddd, doublet of doublet of doublets; dt, doublet of triplets; m, multiplet; quint, quintuplet; sext, sextuplet sept, septet; br, broad signal; dq, doublet of quartets. High-resolution mass spectra (HRMS) were acquired using a Bruker solariX XR Fourier transform ion cyclotron resonance mass spectrometer (Bruker Daltonik GmbH, Bremen, Germany) equipped with a 7 T refrigerated actively shielded superconducting magnet. The samples were ionized in positive-ion mode using a MALDI or ESI ionization source. Melting points were measured with a Stuart Model SMP 30 melting point apparatus and are uncorrected. All starting materials (unless otherwise noted) were purchased from Aldrich and TCI used as received. *N*-SCF_3_ phthalimide **2** and the pyrazolamides **1** were prepared by using general procedures reported in literature.^10^

### Synthesis of *N*-Benzyl α-Trifluoromethylthioamide (**4**) under Batch Conditions

In an oven-dried vial, under nitrogen atmosphere, TEA (5.6 μL 0.04 mmol, 0.2 equiv) was added to a mixture of *N*-acylpyrazole **1** (0.2 mmol, 1 equiv) and *N*-(trifluoromethylthio)phthalimide **2** (59.3 mg, 0.24 mmol, 1.2 equiv) in a mixture of anhydrous CH_3_CN and THF (1:1, 1 mL). The reaction mixture was stirred at room temperature and monitored by TLC. After completion of the first step, *N*-benzylamine (0.26 mmol, 1.3 equiv) was added, and the mixture was stirred for 2 h at room temperature. After completion, the solvent was evaporated, and the crude mixture was purified by flash chromatography (eluent: hexane/ethyl acetate 100/0 to 80/20) to afford products **4** in 70% yield.

### General Flow Reactor Setup

The coil reactor was realized by using PTFE tubing (1.58 mm outer diameter, 0.78 mm inner diameter) coiled in a bundle and immersed in an oil bath heated to the desired temperature. Reagent solutions were mixed using a PEEK T-mixer. The end of tubing chain connected to a 75 psi back-pressure regulator, and the outlet stream was collected in vial. For further details, see the Supporting Information.

### In-Flow Synthesis of Acyl Pyrazole (**1**)

Syringe A: 2.5 mL SGE gastight syringe containing 4-Br-phenylacetic acid (2 mL of a 0.6 M solution in CH_3_CN/THF 1:1, 0.4 mmol, 1.0 equiv) and DMAP (0.06 M solution CH_3_CN/THF 1:1, 0.04 mmol, 0.1 equiv) and Syringe B: 2.5 mL SGE gastight syringe containing 3 phenylpyrazole (2 mL of a 0.63 M solution in CH_3_CN/DCM 1:1, 0.42 mmol, 1.05 equiv) and EDC·HCl (0.72 M solution in CH_3_CN/DCM 1:1, 0.48 mmol, 1.2 equiv), were connected by a PEEK tee junction to a 500 μL PTFE coil reactor. Both syringes fed the solution at 125 μL/min giving a residence time of 2 min. The system was pressurized at 75 PSI by applying a PEEK black pressure regulator. The outcome of the reactor was collected in a vial, and the solvent was evaporated. After the first five volumes were discarded, steady-state conditions were reached. The yield of product was evaluated by ^1^H NMR, using 1,3,5-trimethoxybenzene as an internal standard (0.33 equiv), and was reported as an average value calculated on three separately collected reactor volumes.

### In-Flow Synthesis of *N*-Benzyl α-Trifluoromethylthioamide (**4**)

Syringe A: 5 mL SGE gastight syringe containing pyrazoleamide **1** (3 mL of a 0.3 M solution in CH_3_CN/THF 1:1, 0.9 mmol), Syringe B: 1 mL SGE gastight syringes containing TEA (0.68 M solution in CH_3_CN/THF 1:1, 0.68 mmol), and Syringe C: 1 mL SGE gastight syringes containing *N*-SCF_3_ phtalimide (1.6 M solution in CH_3_CN/THF 1:1, 1.6 mmol) and α,α,α-CF_3_ toluene as an internal standard (1.3 M solution in CH_3_CN/THF 1:1, 1.3 mmol) were connected by a PEEK tee junction to a 500 μL PTFE coil reactor. Syringe A fed the solution at 25 μL/min, while both syringes B and C fed the solution at 5.5 μL/min, giving a residence time of 14 min and a molar ratio in the reactor of pyrazoleamide (1 mol/equiv), TEA (0.5 mol/equiv), *N*-SCF_3_ phtalimide (1.2 mol/equiv), and α,α,α-CF_3_ toluene (1 equiv). The outcome of the reactor was collected in a vial containing benzylamine (0.2 mmol, 2 equiv), where it was stirred for further 2 h. The reaction mixture was dissolved in CDCl_3_ (without removing reaction solvent to avoid the evaporation of the internal standard) and subjected to ^19^F NMR, to evaluate the NMR yield of the product.^11^ After discarding the first reaction volume, steady-state conditions were reached. The yield was reported as an average value calculated on three separately collected reactor volumes. Reactor volumes were reunited and purified by column chromatography.

### Telescoped Synthesis of α-Trifluoromethylthioamides (**4**–**9**)

Syringe A: 5 mL SGE gastight syringe containing the appropriate phenylacetic acid (0.6 M solution in CH_3_CN/THF 1:1, 1.8 mmol, 1 equiv) and DMAP (0.06 M solution CH_3_CN/THF 1:1, 0.18 mmol 0.1 equiv) and Syringe B: 5 mL SGE gastight syringe, containing 3-phenylpyrazole (0.63 M solution in CH_3_CN/DCM 1:1, 1.89 mmol, 1.05 equiv) and EDC·HCl (0.72 M solution in CH_3_CN/DCM 1:1, 2.16 mmol, 1.2 equiv) were connected by a PEEK tee junction to a 50 μL PTFE coil reactor. Both syringes fed the solution at 12.5 μL/min giving a residence time of 2 min. The outcome of the reactor was connected by another tee junction to two 2.5 mL SGE gastight syringes: Syringe C containing TEA (0.68 M solution in CH_3_CN/THF 1:1, 1.70 mmol) and Syringe D containing *N*-SCF_3_ phtalimide **2** (2 M solution in CH_3_CN/THF 1:1, 5.11 mmol) and α,α,α-CF_3_ toluene as an internal standard (1.3 M solution in CH_3_CN/THF 1:1, 3.25 mmol). Both the syringes C and D fed the solution at 5.5 μL/min, giving an overall residence time of 36 min and a molar ratio in the reactor of pyrazoleamide (1 mol/equiv), TEA (0.5 mol/equiv), *N*-SCF_3_ phtalimide (1.5 mol/equiv), and α,α,α-CF_3_ toluene (1 equiv). The system was pressurized at 75 PSI by applying a PEEK black pressure regulator. To avoid the precipitation of the *N*-SCF_3_ reagent, syringe D was kept at 35 °C. After discarding the first volume, the outcome of the reactor was collected in a vial containing the proper amine (0.2 mmol, 2 equiv), and the mixture was stirred for further 2 h at the temperature reported in Figure 1. The reaction mixture was dissolved in CDCl_3_ (without removing the reaction solvent to avoid the evaporation of the internal standard) and subjected to ^19^F NMR, to evaluate the yield of the product. The ^19^F NMR experiments were recorded with d1 = 5 s to obtain a quantitative analysis. The yield was evaluated by the integral ratio between the signal of SCF_3_ group of the product (∼−40 ppm) and the signal of the CF_3_ group of the internal standard (−63 ppm). The NMR yield was reported as an average value calculated on three separately collected reactor volumes. Reactor volumes were reunited and purified by column chromatography.

#### *N*-Benzyl-2-(4-bromophenyl)-2-((trifluoromethyl)thio)acetamide (**4**)

The compound was purified by flash silica gel column chromatography (hexane/ethyl acetate 100/0 to 80/20). White solid, 85.7 mg, 70% yield. Mp: 130.5–131.4 °C. ^1^H NMR (CDCl_3_, 300 MHz): δ 7.53 (d, 2H, *J* = 8.3 Hz), 7.35–7.28 (m, 5H), 7.22 (d, 2H, *J* = 7.5 Hz), 6.26 (br, 1H), 4.99 (s, 1H), 4.52 (dd, 1H, *J* = 14.7 Hz, *J* = 5.7 Hz), 443 (dd, 1H, *J* = 14.7 Hz, *J* = 5.7 Hz). ^13^C{^1^H} NMR (CDCl_3_, 75 MHz): δ 167.1, 137.0, 134.4, 132.4, 129.7, 129.7 (q, ^1^JCF = 308.3 Hz), 128.8, 127.9, 127.7, 123.3, 53.0, 44.4. ^19^F NMR (CDCl_3_, 282 MHz): δ −40.6. HRMS (ESI-FT ICR) *m*/*z*: [M + Na]^+^ Calcd for C_16_H_13_BrF_3_NOSNa 425.9751; Found 425.9759.

#### *N*-Butyl-2-(4-bromophenyl)-2-((trifluoromethyl)thio)acetamide (**5**)

The compound was purified by flash silica gel column chromatography (hexane/ethyl acetate 100/0 to 80/20). White solid, 66.2 mg, 60% yield. Mp: 85.6–87.0 °C. ^1^H NMR (CDCl_3_, 300 MHz): δ 7.52 (d, 2H, *J* = 8.3 Hz), 7.30 (d, 2H, *J* = 8.3 Hz), 6.08 (br, 1H), 4.96 (s, 1H), 3.29 (ddd, 2H, *J* = *J* = *J* ≈ 7.0 Hz), 1.49 (quint, 2H, *J* = 7.3 Hz), 1.31 (sext, 2H, *J* = 7.4 Hz), 0.92 (t, 3H, *J* = 7.3 Hz). ^13^C{^1^H} NMR (CDCl_3_, 62.5 MHz): δ 167.0, 134.5, 132.3, 129.74 (q, ^1^JCF = 308.4 Hz), 129.7, 123.1, 52.9, 40.0, 31.2, 19.9, 13.6. ^19^F NMR (CDCl_3_, 376 MHz): δ −40.6. HRMS (MALDI-FT ICR) *m*/*z*: [M + H]^+^ Calcd for C_13_H_16_BrF_3_NOS 370.0083; Found 370.0094.

#### *N-*Benzyl-2-(4-chlorophenyl)-2-((trifluoromethyl)thio)acetamide (**6**)

The compound was purified by flash silica gel column chromatography (hexane/ethyl acetate 100/0 to 80/20). White solid, 57.8 mg, 55% yield. Mp: 127.8–129.1 °C. ^1^H NMR (CDCl_3_, 400 MHz): δ 7.33–7.29 (m, 5H), 7.31 (d, 2H, *J* = 7.2 Hz), 7.17 (d, 2H, *J* = 7.2 Hz), 6.51 (br, 1H), 5.01 (s, 1H), 4.45 (dd, 1H, *J* = 14.8 Hz, *J* = 5.8 Hz), 4.37 (dd, 1H, *J* = 14.8 Hz, *J* = 5.8 Hz). ^13^C{^1^H} NMR (CDCl_3_, 100 MHz): δ 167.1, 137.0, 135.1, 133.8, 129.7 (q, ^1^JCF = 308.3 Hz), 129.4 (2C), 128.8, 127.9, 127.7, 52.9, 44.3. ^19^F NMR (CDCl_3_, 376 MHz): δ −40.6. HRMS (MALDI-FT ICR) *m*/*z*: [M + H]^+^ Calcd for C_16_H_14_ClF_3_NOS 360.0437; Found 360.0431.

#### *N*-Butyl-2-(4-chlorophenyl)-2-((trifluoromethyl)thio)acetamide (**7**)

The compound was purified by flash silica gel column chromatography (hexane/ethyl acetate 100/0 to 80/20). White solid, 49.4 mg, 50% yield. Mp: 72.7–73.7 °C. ^1^H NMR (CDCl_3_, 300 MHz): δ 7.36 (m, 4H), 6.24 (br, 1H), 4.99 (s, 1H), 3.28 (ddd, 2H, *J* = *J* = J ≈ 7.0 Hz), 1.48 (quint, 2H, *J* = 7.4 Hz), 1.30 (sext, 2H, *J* = 7.4 Hz), 0.9 (t, 3H, *J* = 7.3 Hz). ^13^C{^1^H} NMR (CDCl_3_, 75 MHz): δ 167.2, 135.0, 134.0, 129.80 (q, ^1^JCF = 308.2 Hz), 129.4, 129.3, 52.9, 40.1, 31.2, 19.9, 13.6. ^19^F NMR (CDCl_3_, 376 MHz): δ −40.7. HRMS (ESI-FT ICR) *m*/*z*: [M + H]^+^ Calcd for C_13_H_16_ClF_3_NOS 326.0588; Found 326.0574.

#### *N*-Benzyl-2-(2-nitrophenyl)-2-((trifluoromethyl)thio)acetamide (**8**)

The compound was purified by flash silica gel column chromatography (hexane/ethyl acetate 100/0 to 80/20). Colorless oil, 57.2 mg, 51% yield. ^1^H NMR (CDCl_3_, 400 MHz): δ 8.07 (d, 1H, *J* = 1.4 Hz), 7.76–7.68 (m, 2H), 7.55 (t, 1H, *J* = 7.7 Hz), 7.35–7.22 (m, 5H), 7.00 (br, 1H), 5.45 (s,1H), 4.54 (dd, 1H, *J* = 14.9 Hz, *J* = 5.9 Hz), 4.41 (dd, 1H, *J* = 14.9 Hz, *J* = 5.7 Hz). ^13^C{^1^H} NMR (CDCl_3_, 150 MHz): δ 166.1, 147.5, 137.2, 134.5, 132.8, 132.2, 130.3 (q, ^1^JCF = 308.4 Hz), 129.9, 128.9, 127.9, 127.7, 125.4, 49.7, 44.6. ^19^F NMR (CDCl_3_, 376 MHz): δ −41.5. HRMS (MALDI-FT ICR) *m*/*z*: [M + Na]^+^ Calcd for C_16_H_13_F_3_N_2_O_3_SNa 393.0497, Found 393.0472.

#### *N,N*-Diethyl-2-(4-bromophenyl)-2-((trifluoromethyl)thio)acetamide (**9**)

The compound was purified by flash silica gel column chromatography (hexane/ethyl acetate 100/0 to 80/20). Pale yellow oil, 33.6 mg, 30% yield. °C. ^1^H NMR (CDCl_3_, 400 MHz): δ 7.50 (d, 2H, *J* = 8.4 Hz), 7.34 (d, 2H, *J* = 8.4 Hz), 5.28 (s, 1H), 3.41 (m, 1H), 3.31 (m, 2H), 3.21 (m, 1H), 1.08 (t, 3H, *J* = 7.2 Hz), 1.02 (t, 3H, *J* = 7.0 Hz). ^13^C{^1^H} NMR (CDCl_3_, 100 MHz): δ 166.5, 135.7, 132.3, 130.6 (q, ^1^JCF = 308.1 Hz), 129.8, 122.9, 51.9, 42.5, 41.0, 14.0, 12.5. ^19^F NMR (CDCl_3_, 376 MHz): δ −40.4. HRMS (ESI-FT ICR) *m*/*z*: [M + H]^+^ Calcd for C_13_H_15_BrF_3_NOS 370.0083, Found 370.0087.

### Telescoped Synthesis of Ethyl 2-(4-Bromophenyl)-2-((trifluoromethyl)thio)acetate (**10**)

Syringe A: 5 mL SGE gastight syringe containing 4-Br-phenylacetic acid (0.6 M solution in CH_3_CN/THF 1:1, 1.8 mmol, 1.0 equiv) and DMAP (0.06 M solution CH_3_CN/THF 1:1, 0.18 mmol, 0.1 equiv) and Syringe B: 5 mL SGE gastight syringe containing 3-phenylpyrazole (0.63 M solution in CH_3_CN/DCM 1:1, 1.89 mmol, 1.05 equiv) and EDC·HCl (0.72 M solution in CH_3_CN/DCM 1:1, 2.16 mmol, 1.2 equiv) were connected by a PEEK tee junction to a 50 μL PTFE coil reactor. Both syringes fed the solution at 12.5 μL/min giving a residence time of 2 min. The outcome of the reactor was connected by another tee junction to two 2.5 mL SGE gastight syringes: Syringe C containing TEA (0.68 M solution in CH_3_CN/THF 1:1, 1.70 mmol) and Syringe D containing *N*-SCF3 phtalimide (**2**) (2 M solution in CH_3_CN/THF 1:1, 5.11 mmol) and α,α,α-CF_3_ toluene as an internal standard (1.3 M solution in CH_3_CN/THF 1:1, 3.25 mmol). Both syringes C and D fed the solution at 5.5 μL/min, giving an overall residence time of 36 min and a molar ratio in the reactor of pyrazoleamide (1 mol/eq), TEA (0.5 mol/equiv), *N*-SCF_3_ phtalimide (1.5 mol/equiv), and α,α,α-CF_3_ toluene (1 equiv). The system was pressurized at 75 PSI by applying a PEEK black pressure regulator. To avoid the precipitation of the *N*-SCF_3_ reagent, syringe D was kept at 35 °C. The outcome of the reactor was collected in a vial containing ethanol (5 mmol, 50 equiv) and DMAP (1.3 mg, 0.01 mmol, 0.1 equiv), and the mixture was stirred for a further 8 h at 50 °C. The reaction mixture was dissolved in CDCl_3_ (without removing the reaction solvent to avoid the evaporation of the internal standard) and subjected to ^19^F NMR to evaluate the yield of the product. The ^19^F NMR experiments were recorded with d1 = 5 s to obtain a quantitative analysis. The yield was evaluated by the integral ratio between the signal of SCF_3_ group of the product (∼−40 ppm) and the signal of the CF_3_ group of the internal standard (−63 ppm). The NMR yield was reported as an average value calculated on three separately collected reactor volumes. Reactor volumes were reunited and purified by column chromatography (eluent: hexane/ethyl acetate 100/0 to 90/10). Pale yellow oil, 72.8 mg, 70% yield. ^1^H NMR (CDCl_3_, 300 MHz):7.51 (d, 2H, *J* = 8.3 Hz), 7.33 (d, 2H, *J* = 8.3 Hz), 5.00 (s, 1H), 4.25 (dq, 1H, *J* = 15.8 Hz, *J* = 7.1 Hz), 4.19 (dq, 1H, *J* = 15.8 Hz, *J* = 7.1 Hz), 1.25 (t, 3H, *J* = 7.1 Hz). ^13^C{^1^H} NMR (CDCl_3_, 75 MHz): δ 168.4, 133.3, 132.2, 129.8, 129.6 (q, ^1^JCF = 306.9 Hz), 123.3, 62.8, 50.9, 13.8. ^19^F NMR (CDCl_3_, 376 MHz): δ −41.0. HRMS (ESI-FT ICR) *m*/*z*: [M + Na]^+^ Calcd for C_11_H_10_BrF_3_O_2_SNa 364.9408, Found 364.9429.

## Supporting Information Available

Flow setup for synthesis of α-trifluoromethylthioamide **4** and telescoped synthesis of α-trifluoromethylthio carboxylic acid derivatives. Copies of ^1^H NMR and ^13^C NMR spectra for all compounds (PDF)

## Dedication

Dedicated to Prof. Placido Neri (Università di Salerno) on the occasion of his 60th birthday.
