# Supplementary material for: Continuous Flow Synthesis of α-Trifluoromethylthiolated Esters and Amides from Carboxylic Acids: a Telescoped Approach
Source: J Org Chem. 2021 Jul 27;86(20):14207–12. doi: 10.1021/acs.joc.1c01270 (PMC8524418; doi:10.1021/acs.joc.1c01270)

Supporting Information

Continuous Flow Synthesis of α-Trifluoromethylthiolated Esters and Amides from Carboxylic Acids: a Telescoped Approach

Francesca Franco,† Sara Meninno,† Alessandra Lattanzi,*† Alessandra Puglisi,‡ Maurizio Benaglia*‡

†Dipartimento di Chimica e Biologia "A. Zambelli", Università di Salerno, Via Giovanni Paolo II, 84084, Fisciano, Italy

‡Dipartimento di Chimica, Università degli Studi di Milano, Via Golgi 19, 20133, Milano, Italy

Table of contents

Flow setup for synthesis of *N*-benzyl α-trifluoromethylthio amide 4 .... S2

Flow setup for telescopic synthesis of amides and esters S3

NMR Spectra S4

Flow setup for synthesis of *N*-benzyl α-trifluoromethylthio amide 4

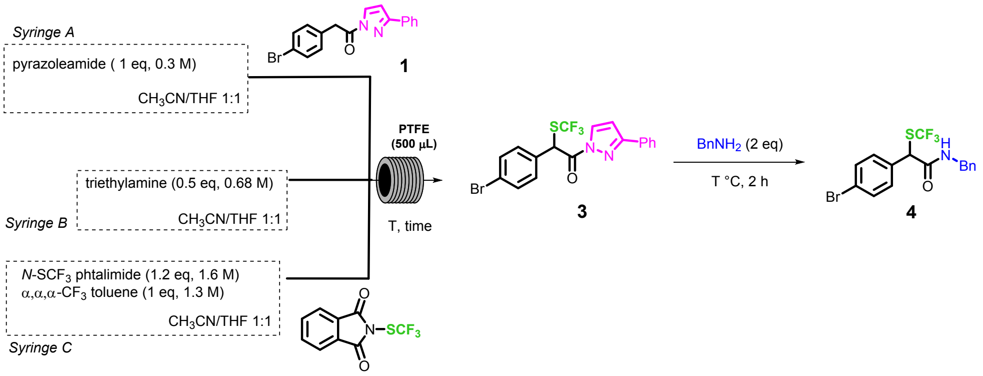

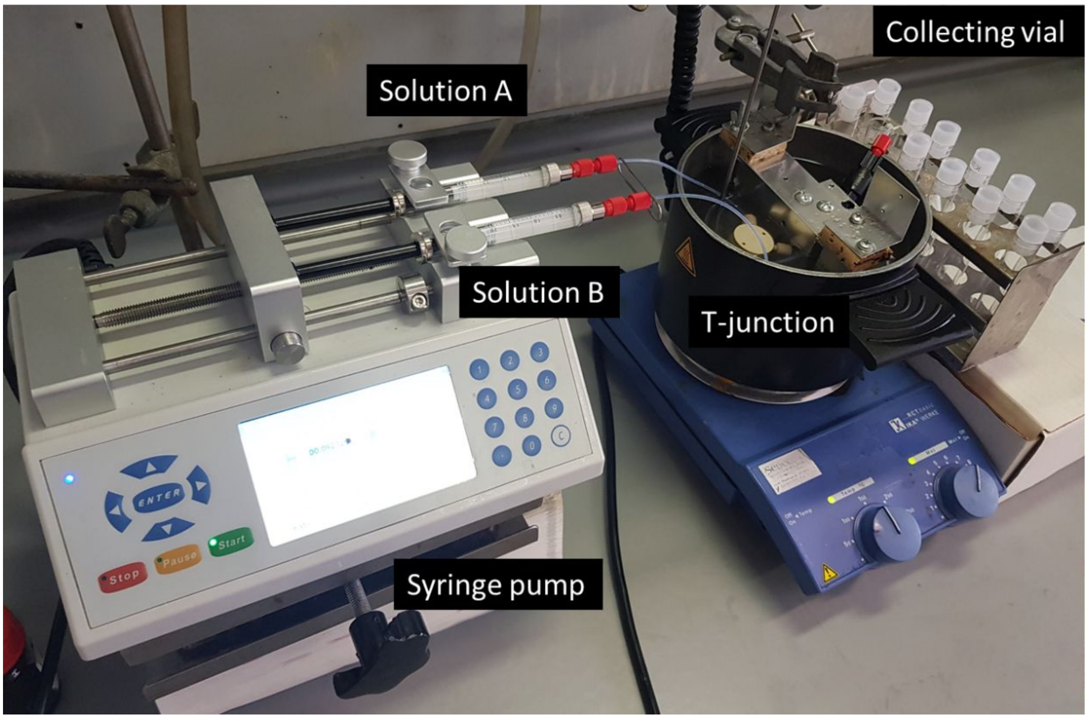

Flow setup for telescopic synthesis of amides and esters

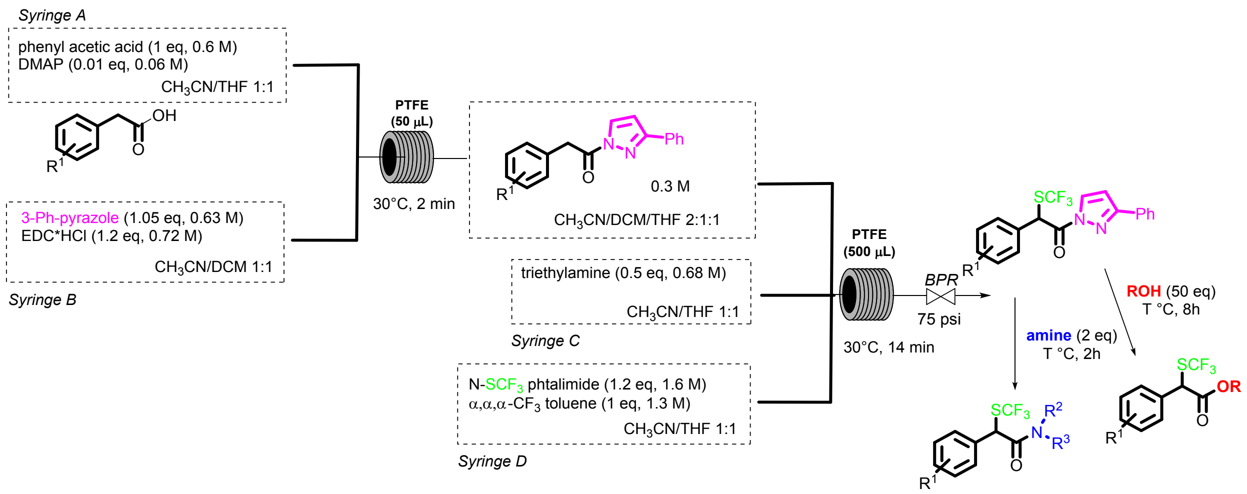

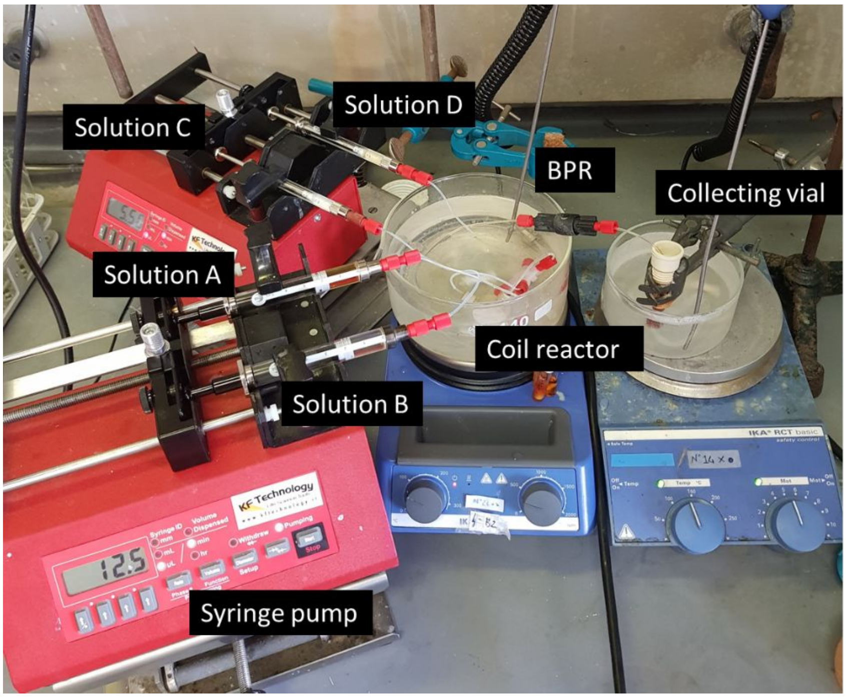

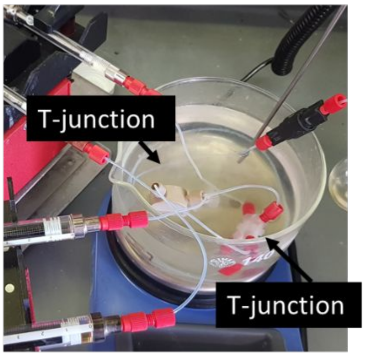

NMR Spectra

^{1}H NMR in $CDCl_3$ of **4** (300 MHz)

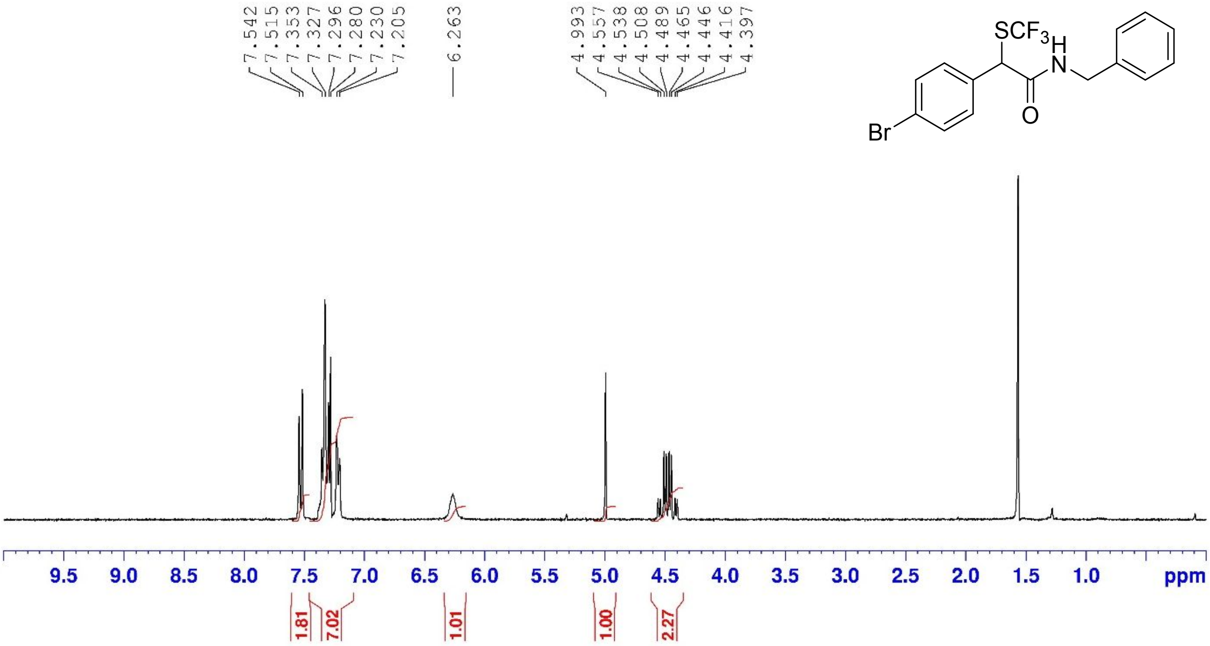

$^{13}C\{^1H\}$ NMR in $CDCl_3$ of **4** (75 MHz)

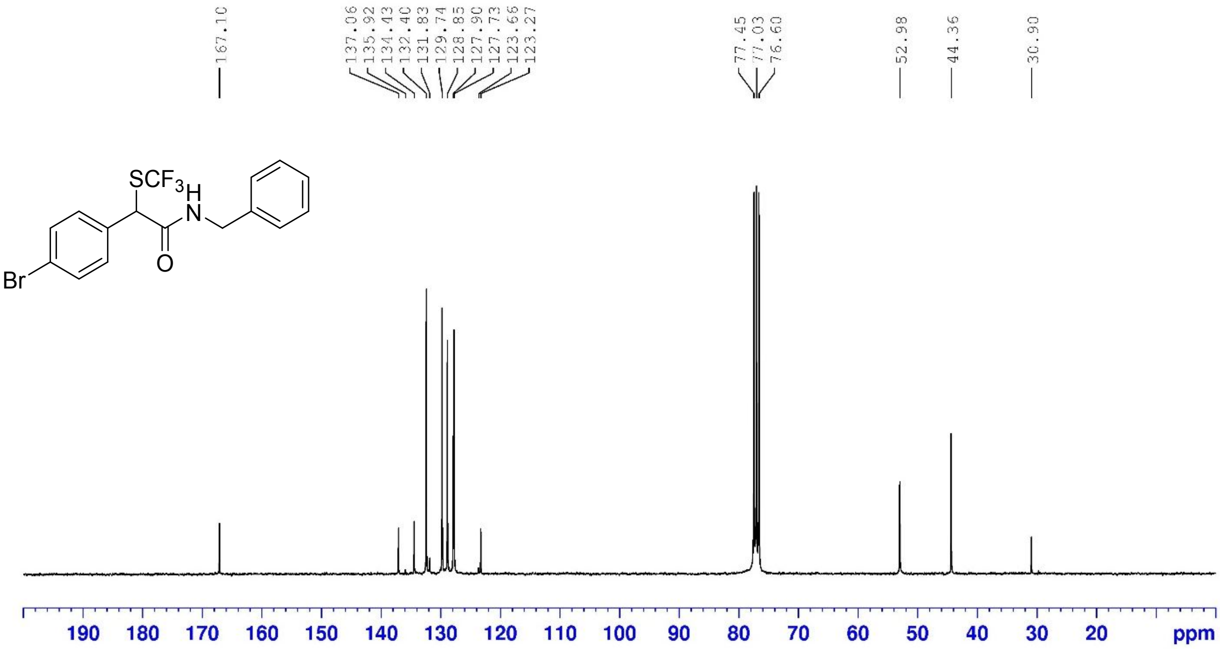

^{19}F NMR in $CDCl_3$ of **4** (282 MHz)

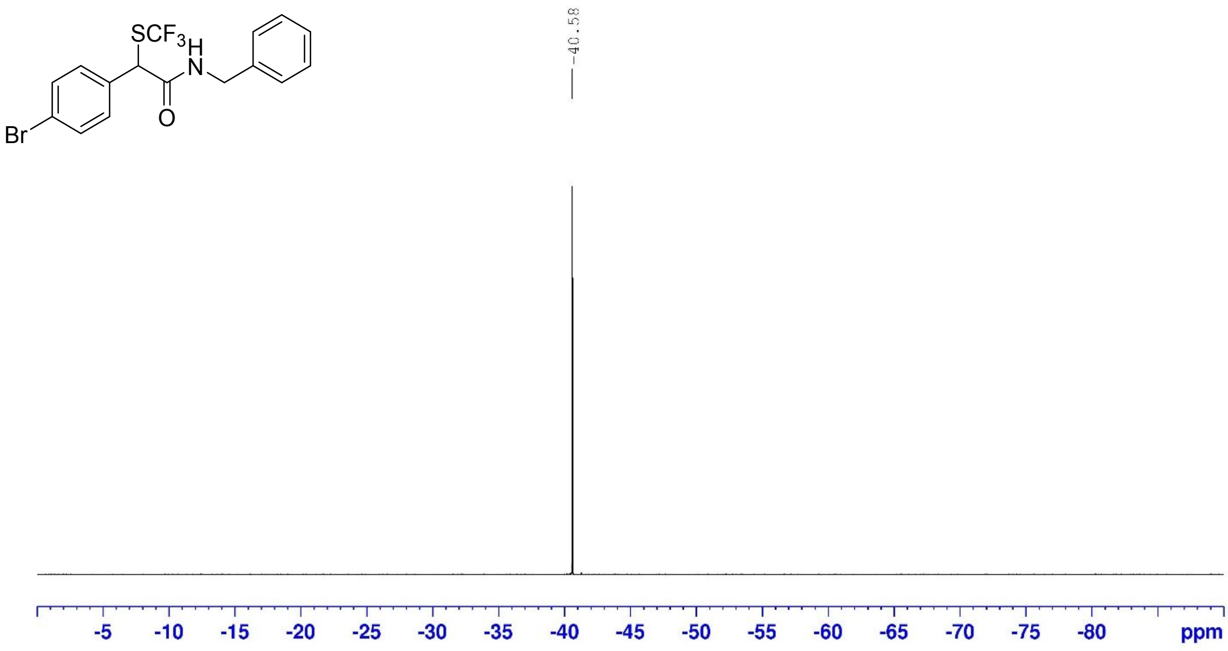

^{1}H NMR in $CDCl_3$ of **5** (300 MHz)

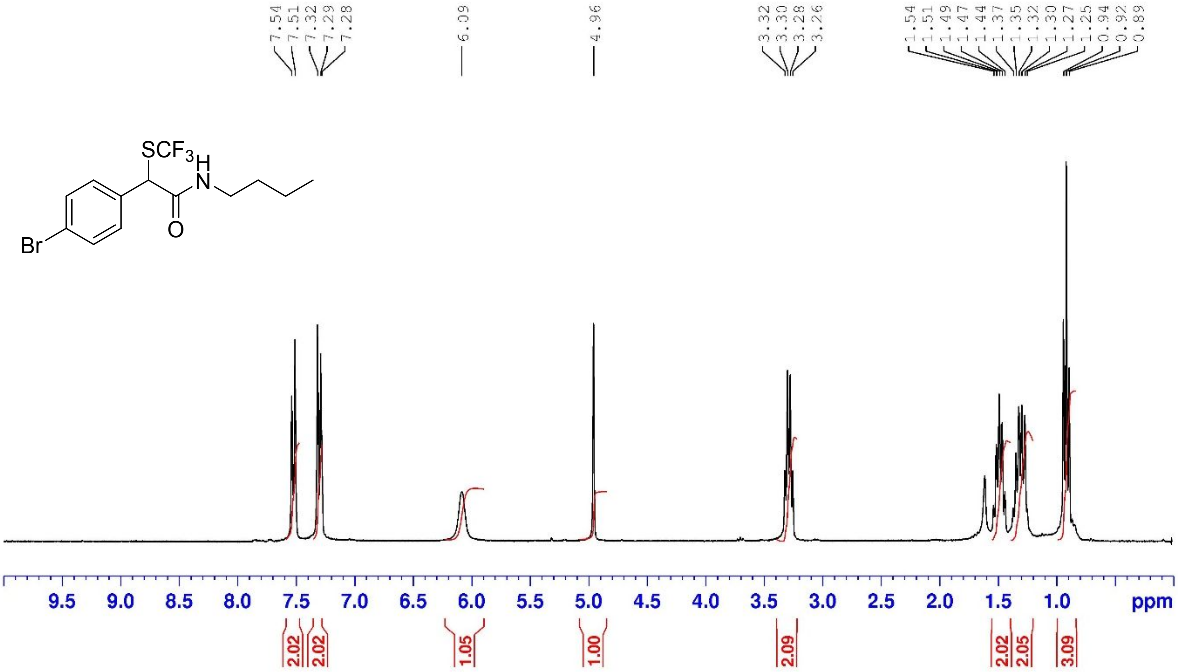

$^{13}C\{^1H\}$ NMR in $CDCl_3$ of **5** (62.5 MHz)

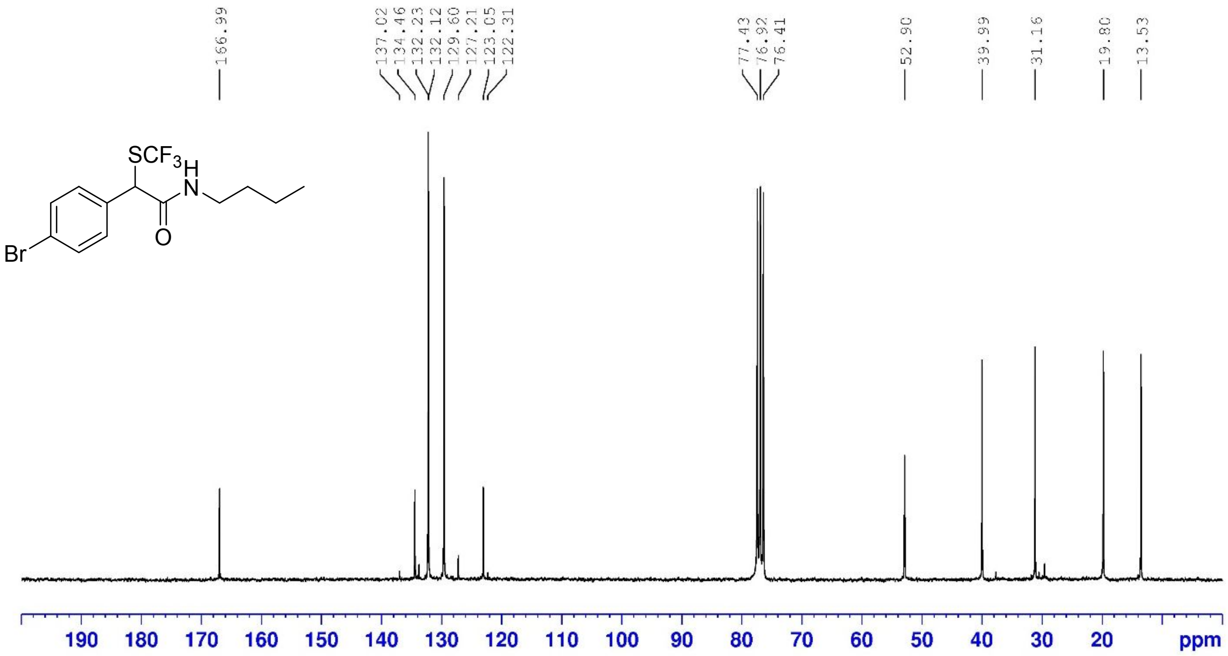

^{19}F NMR in $CDCl_3$ of **5** (376 MHz)

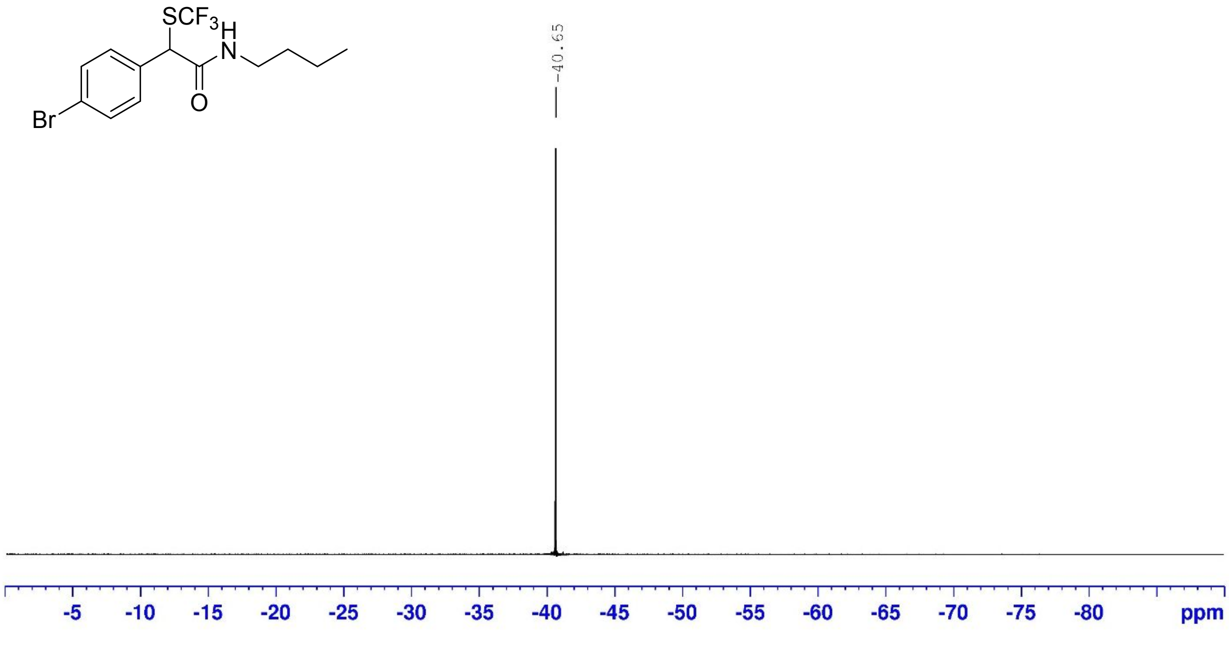

^{1}H NMR in $CDCl_3$ of **6** (400 MHz)

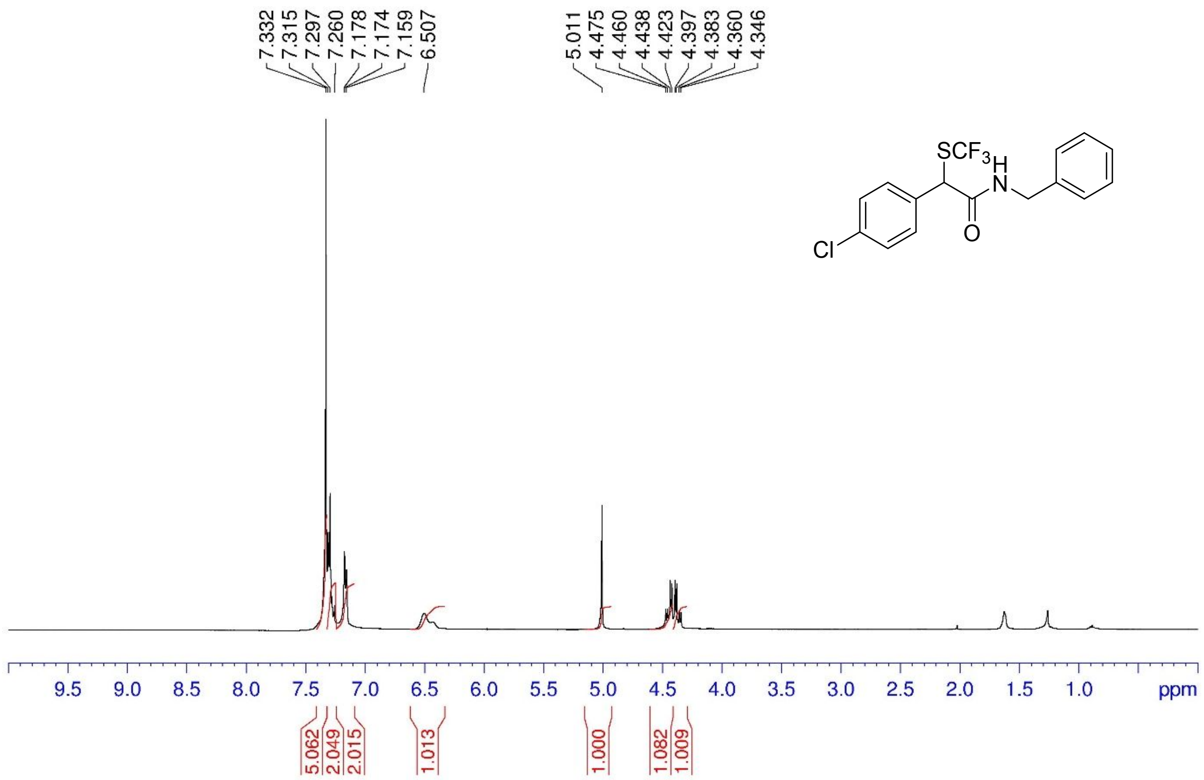

^{13}C{^{1}H} NMR in $CDCl_3$ of **6** (100 MHz)

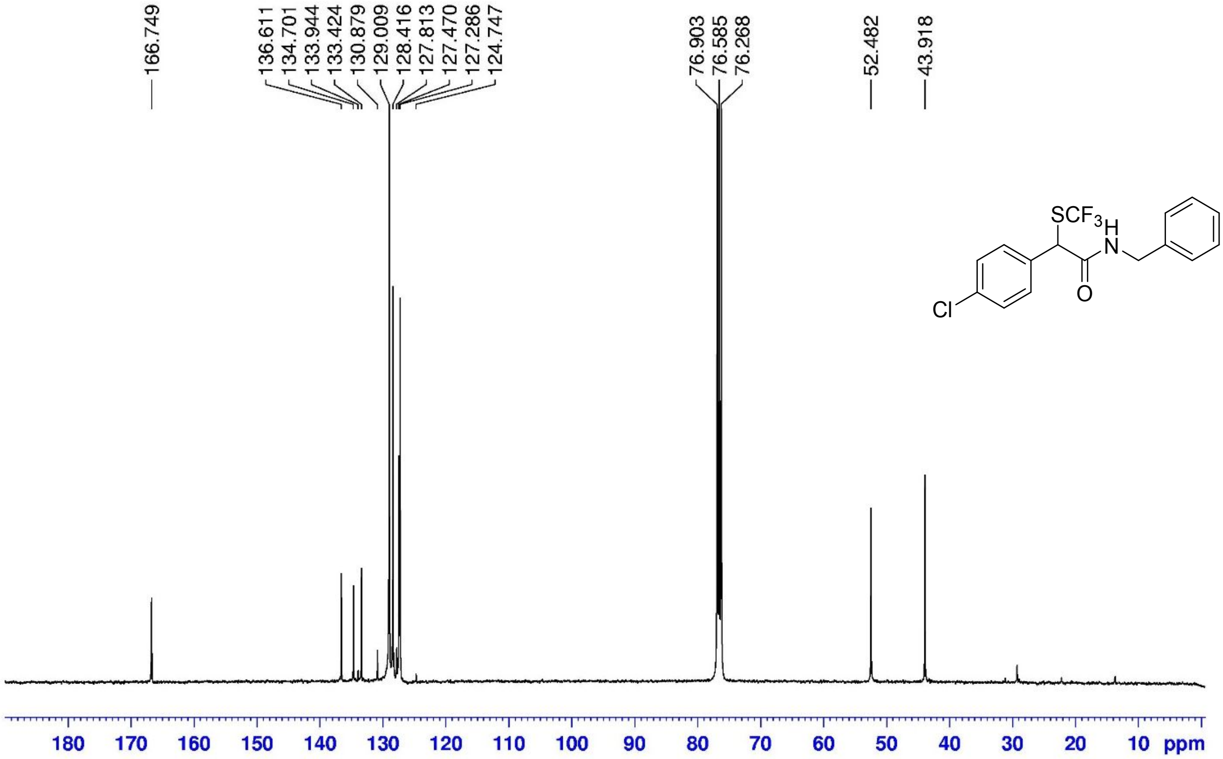

^{19}F NMR in $CDCl_3$ of **6** (376 MHz)

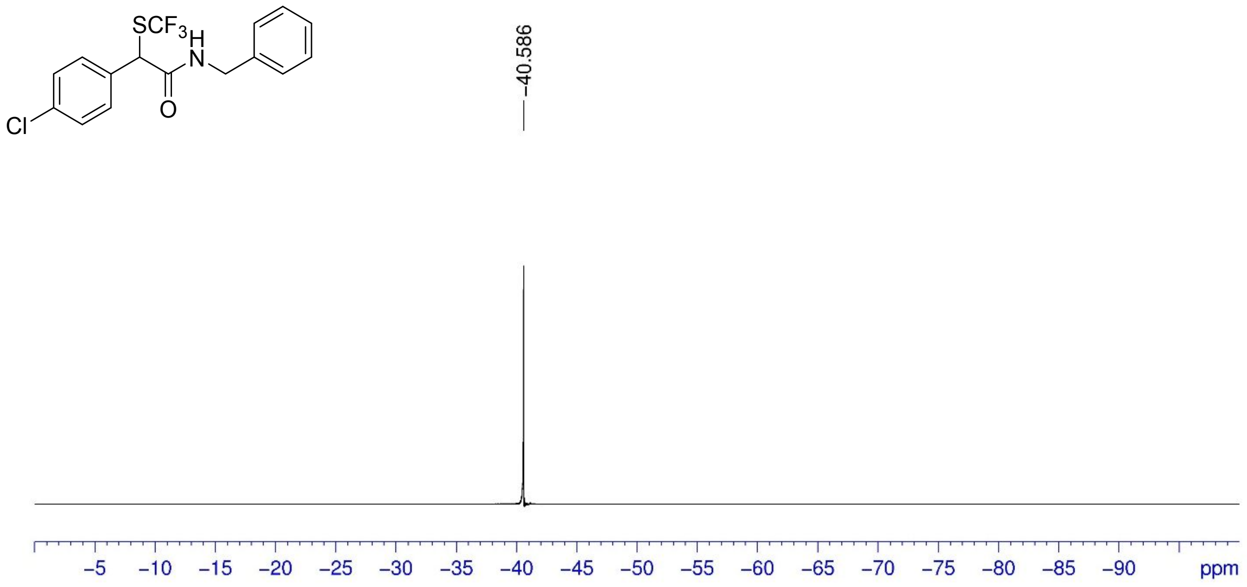

^{1}H NMR in $CDCl_3$ of **7** (300 MHz)

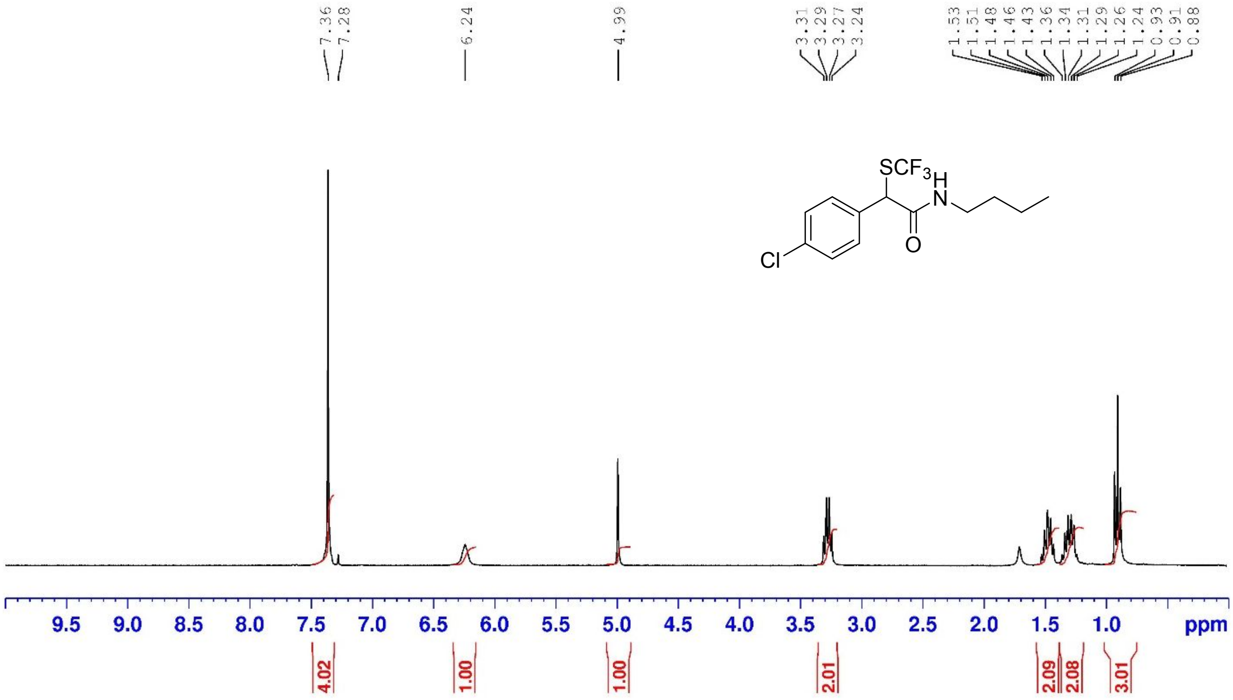

$^{13}C\{^1H\}$ NMR in $CDCl_3$ of **7** (75 MHz)

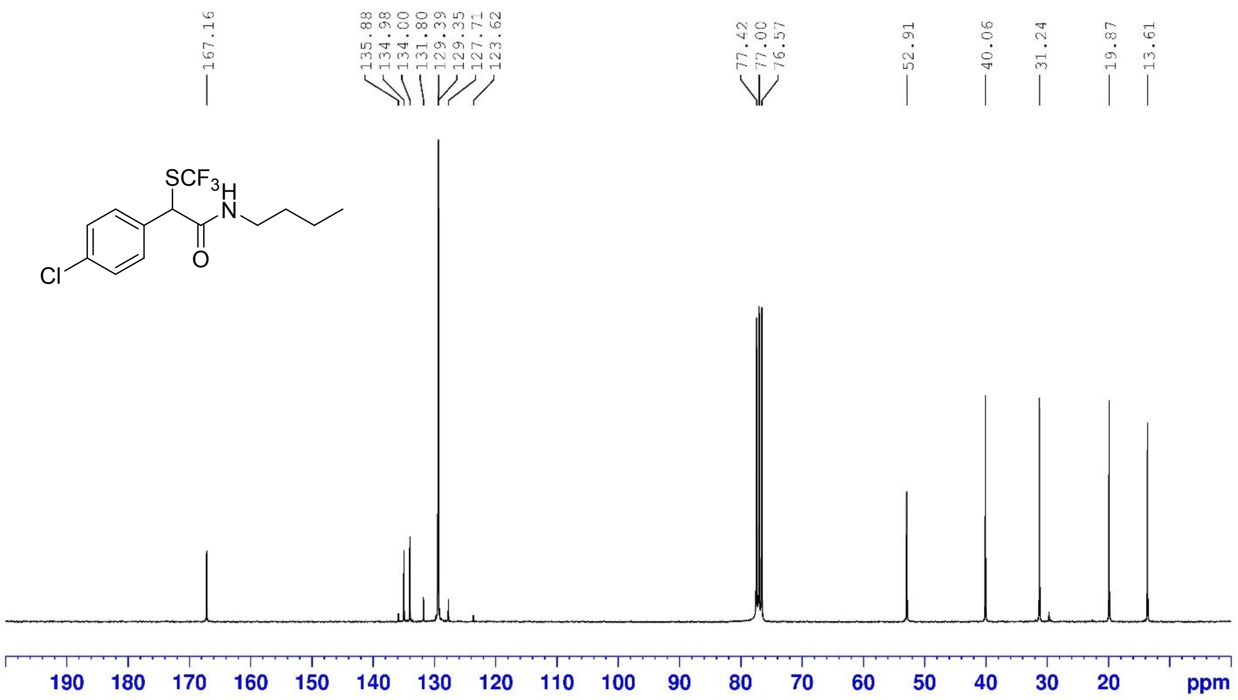

^{19}F NMR in $CDCl_3$ of **7** (376 MHz)

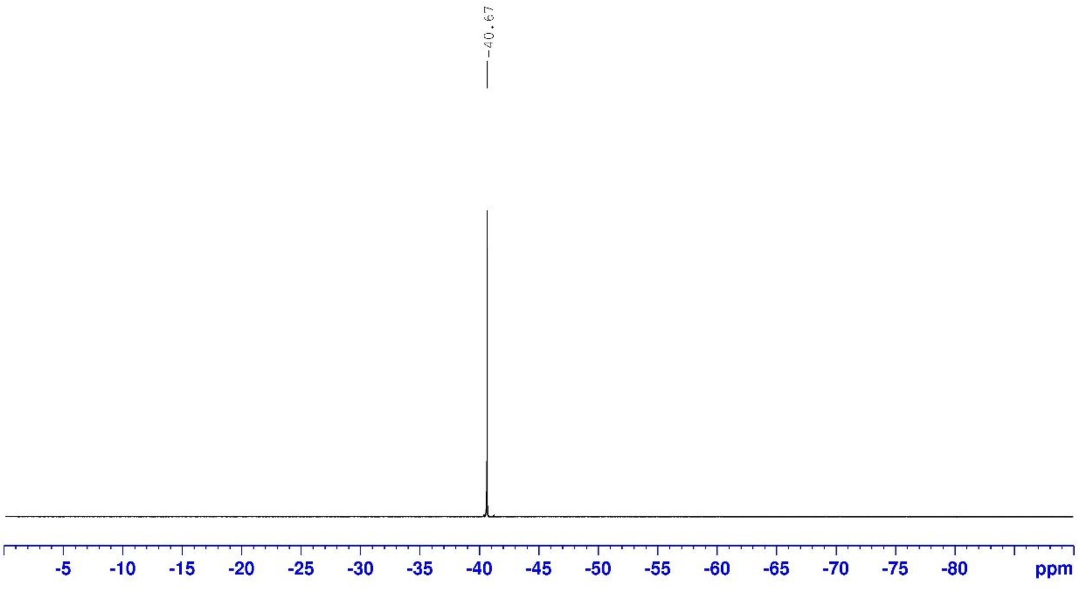

^{1}H NMR in $CDCl_3$ of **8** (400 MHz)

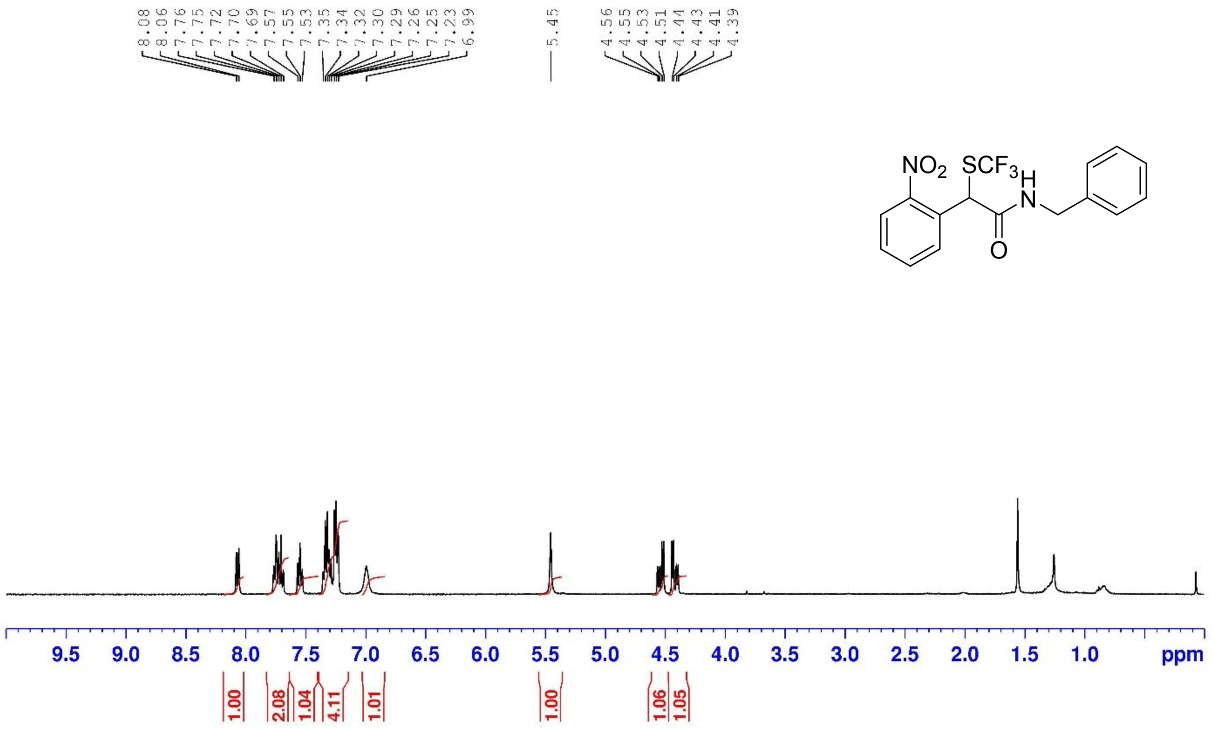

$^{13}C\{^{1}H\}$ NMR in $CDCl_3$ of **8** (150 MHz)

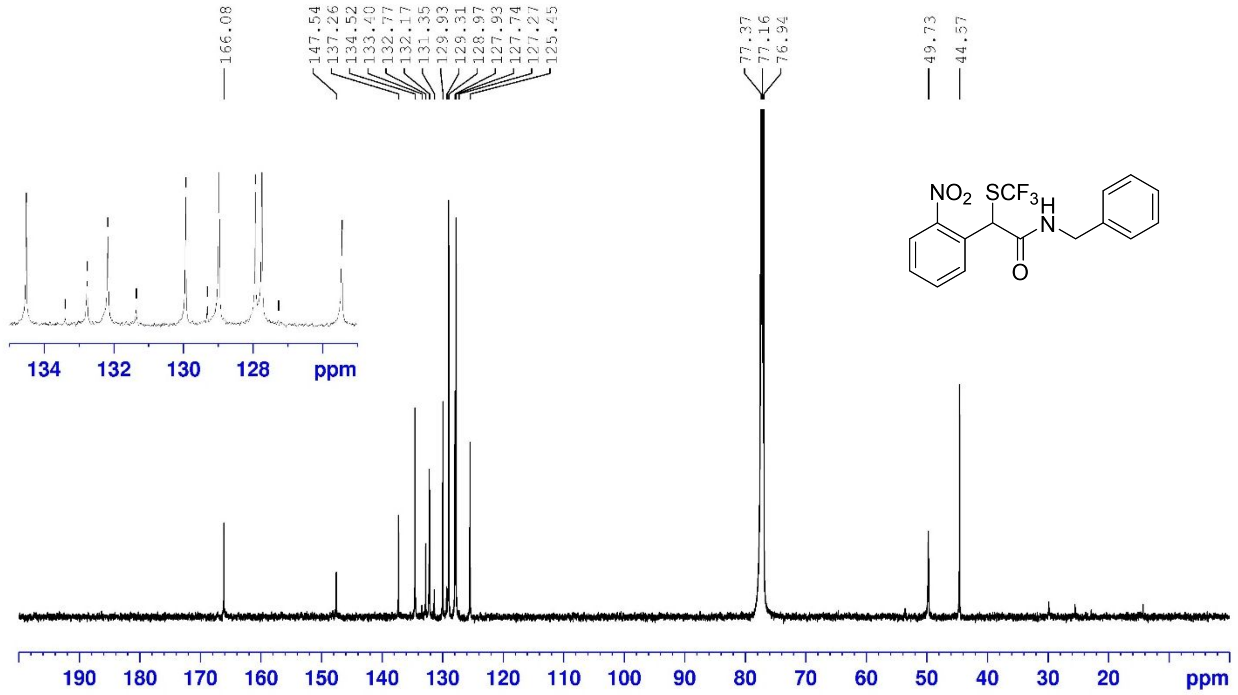

^{19}F NMR in $CDCl_3$ of **8** (376 MHz)

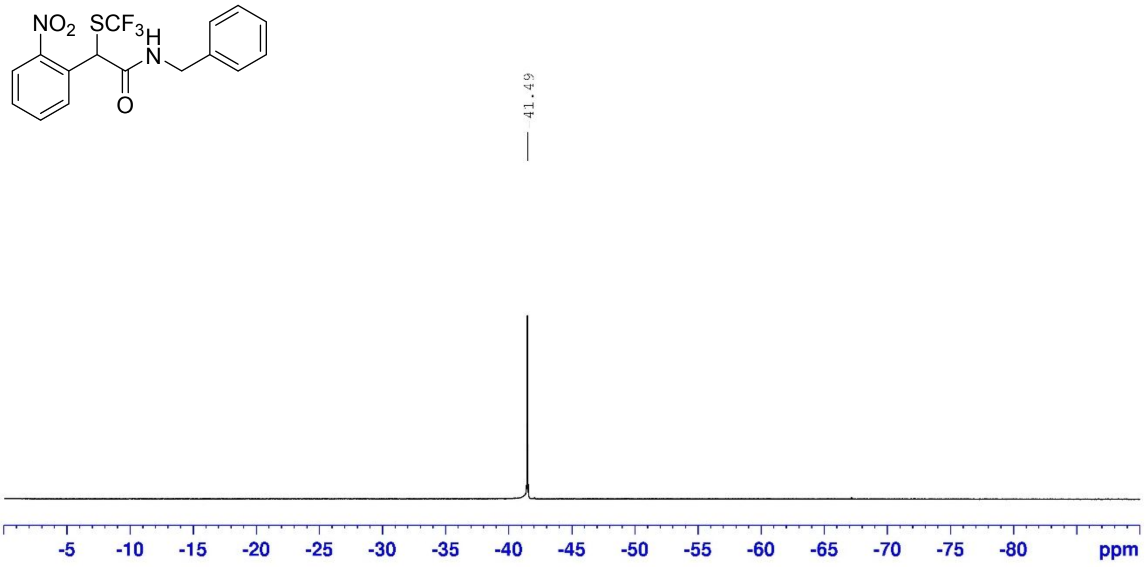

^{1}H NMR in $CDCl_3$ of **9** (400 MHz)

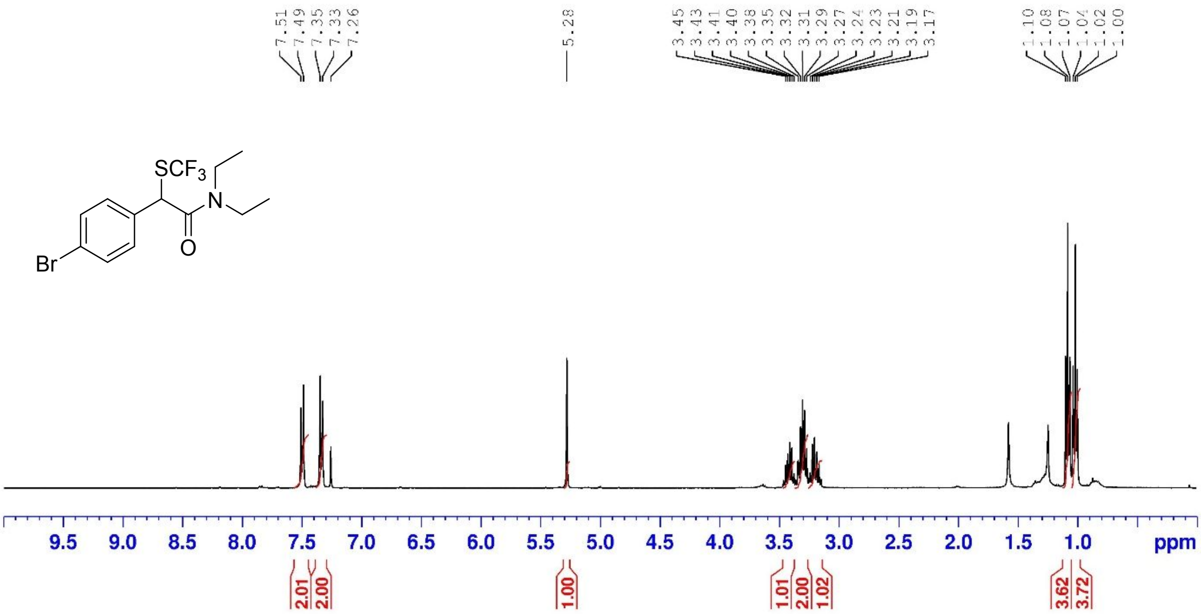

$^{13}C\{^1H\}$ NMR in $CDCl_3$ of **9** (100 MHz)

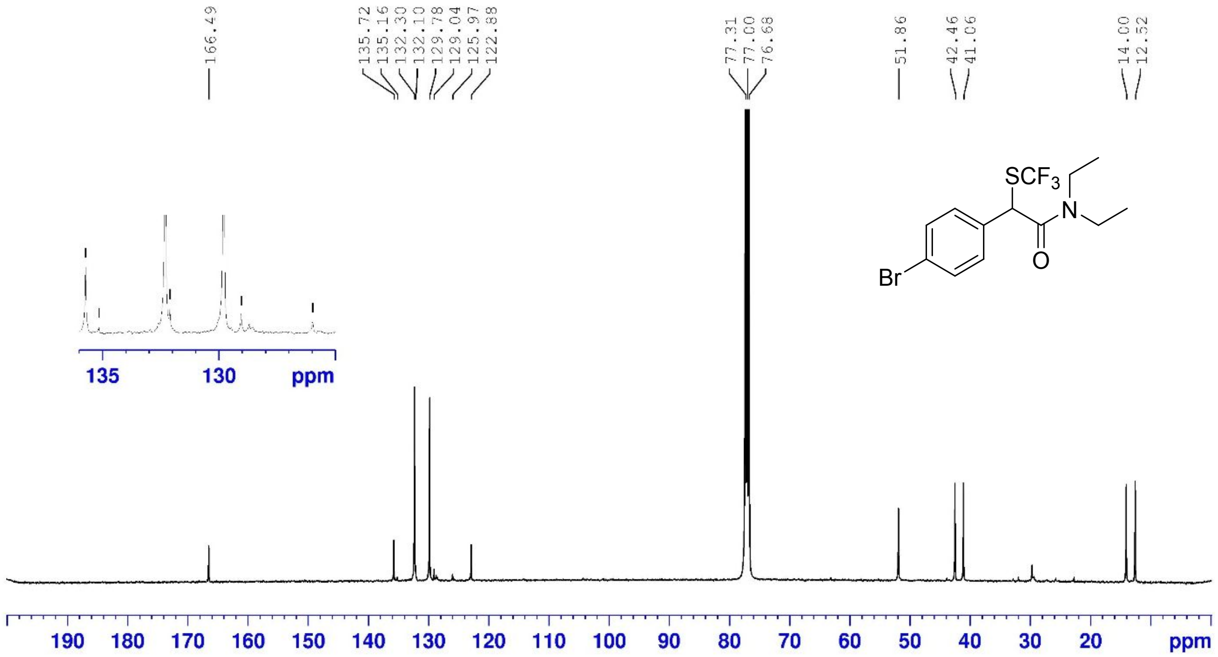

^{19}F NMR in $CDCl_3$ of **9** (376 MHz)

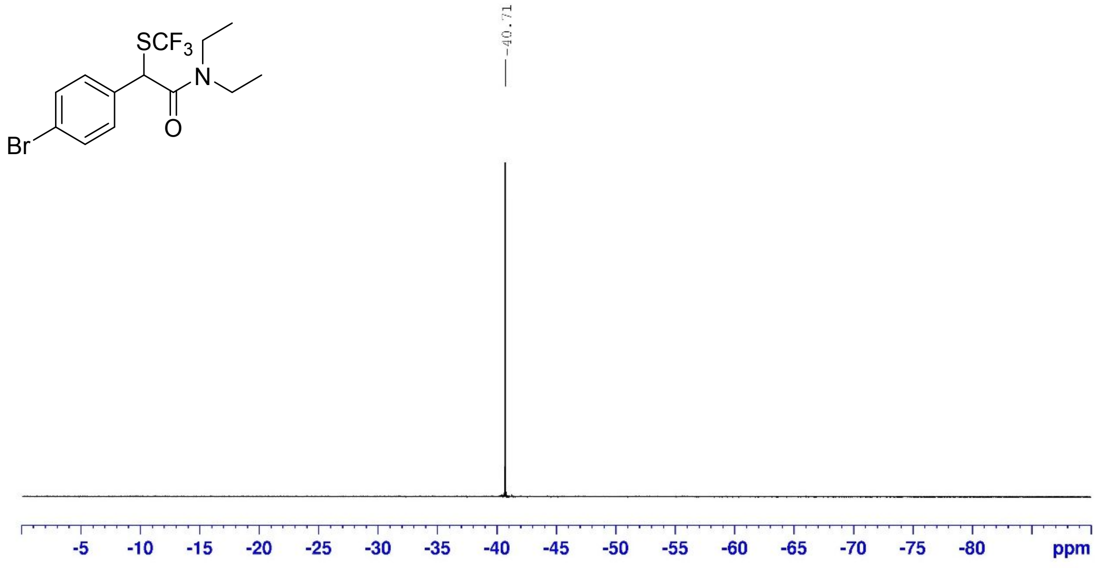

^{1}H NMR in $CDCl_3$ of **10** (300 MHz)

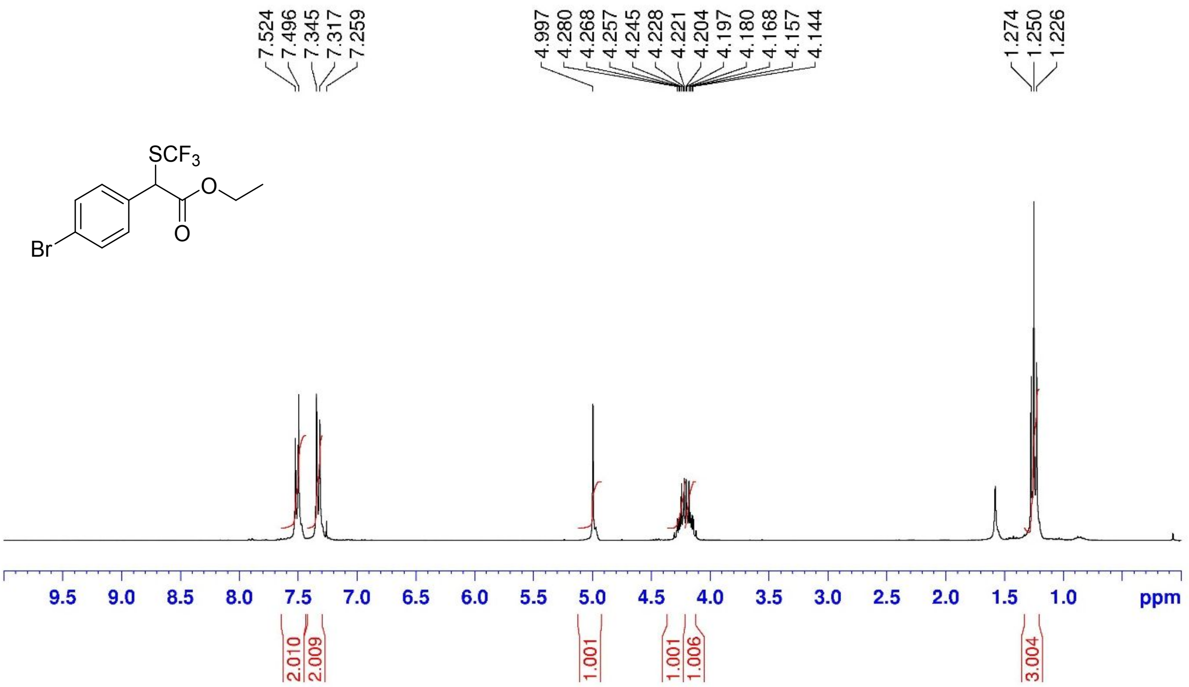

^{13}C{^{1}H} NMR in $CDCl_3$ of **10** (75 MHz)

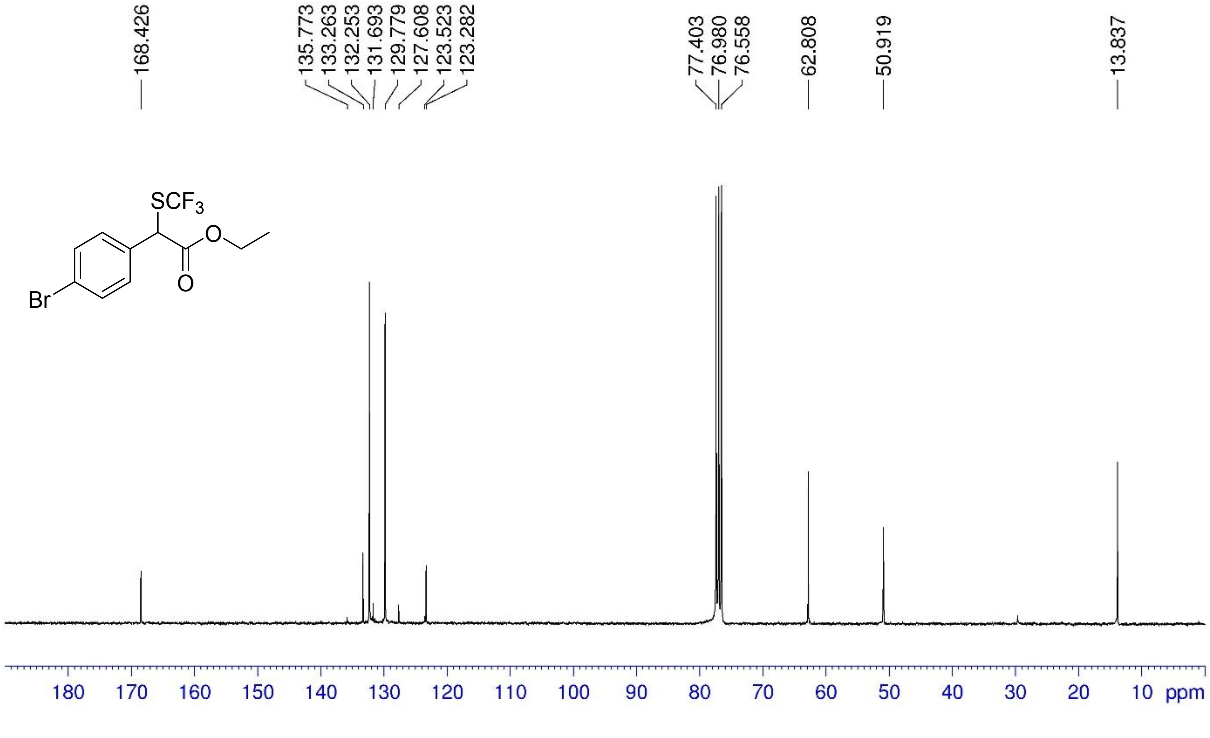

^{19}F NMR in $CDCl_3$ of **10** (376 MHz)

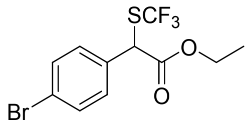

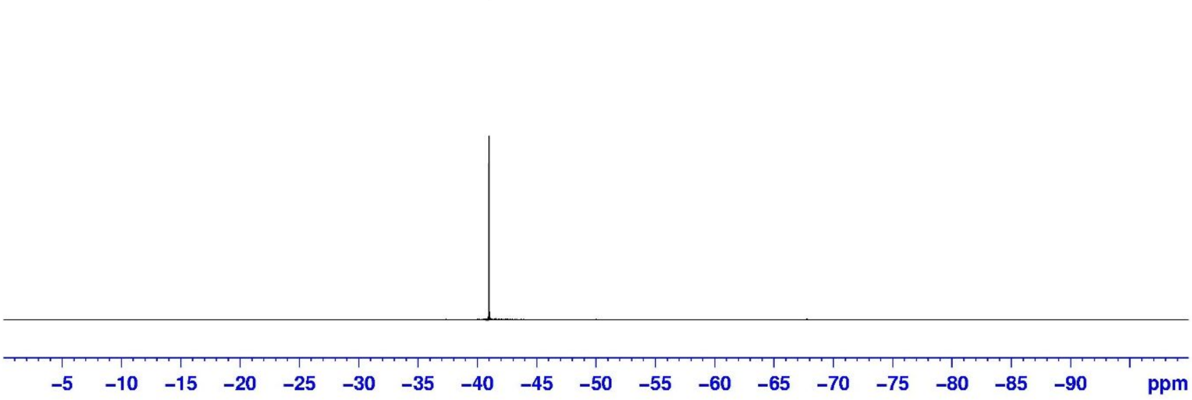

Supplement: Supplementary file 1 — jo1c01270_si_001.pdf [file jo1c01270_si_001.pdf]
